# Primary Mode of Action of the Novel Sulfonamide Fungicide against *Botrytis cinerea* and Field Control Effect on Tomato Gray Mold

**DOI:** 10.3390/ijms23031526

**Published:** 2022-01-28

**Authors:** Xiaojing Yan, Shuning Chen, Wei Sun, Xiaoxin Zhou, Daibin Yang, Huizhu Yuan, Daoquan Wang

**Affiliations:** 1 Institute of Plant Protection, Chinese Academy of Agricultural Sciences, No. 2 Yuanmingyuan West Road, Haidian District, Beijing 100193, China; yanxiaojing@caas.cn (X.Y.); chenshuning@caas.cn (S.C.); 82101205222@caas.cn (W.S.); m13898156865@163.com (X.Z.); yangdaibin@caas.cn (D.Y.); 2 Department of Applied Chemistry, China Agricultural University, No. 2 Yuanmingyuan West Road, Haidian District, Beijing 100193, China

**Keywords:** sulfonamide fungicide, *Botrytis cinerea*, mode of action, field control, tomato gray mold

## Abstract

*Botrytis cinerea* is considered an important plant pathogen and is responsible for significant crop yield losses. With the frequent application of commercial fungicides, *B*. *cinerea* has developed resistance to many frequently used fungicides. Therefore, it is necessary to develop new kinds of fungicides with high activity and new modes of action to solve the increasingly serious problem of resistance. During our screening of fungicide candidates, one novel sulfonamide compound, *N*-(2-trifluoromethyl-4-chlorphenyl)-2-oxocyclohexyl sulfonamide (**L13**), has been found to exhibit good fungicidal activity against *B. cinerea*. In this work, the mode of action of **L13** against *B. cinerea* and the field control effect on tomato gray mold was studied. **L13** had good control against *B. cinerea* resistant to carbendazim, diethofencarb, and iprodione commercial fungicides in the pot culture experiments. SEM and TEM observations revealed that **L13** could cause obvious morphological and cytological changes to *B. cinerea*, including excessive branching, irregular ramification or abnormal configuration, and the decomposition of cell wall and vacuole. **L13** induced more significant electrolyte leakage from hyphae than procymidone as a positive control. **L13** had only a minor effect on the oxygen consumption of intact mycelia, with 2.15% inhibition at 50 μg/mL. In two locations over 2 years, the field control effect of **L13** against tomato gray mold reached 83% at a rate of 450 g ai ha^−1^, better than the commercial fungicide of iprodione. Moreover, toxicological tests demonstrated the low toxicological effect of **L13**. This research seeks to provide technical support and theoretical guidance for **L13** to become a real commercial fungicide.

## 1. Introduction

*Botrytis cinerea* Pers ex Fr (teleomorph: *Botryotinia fuckeliana*) is one of the most commonly studied polyphagous fungal plant pathogens, causing gray mold on more than 1400 known hosts in 586 plant genera and 152 botanical families worldwide [1,2]. Due to both its scientific and economic importance, *B*. *cinerea* is considered among the most important plant pathogens and is responsible for significant crop yield losses [3]. To date, the principal strategy to control gray mold rot caused by *B. cinerea* has been to rely on chemical control through the application of synthetic fungicides [4]. Most of the registered fungicides to control gray mold are quinone outside inhibitors, benzimidazole, carbamates, phenylpyrroles, and succinate dehydrogenase inhibitors [5]. However, because of its host diversity, prolific reproduction, short life cycle, and high genetic variability, during the past decades, many strains of *B. cinerea* with multiple resistance to conventional fungicides have appeared on various crops worldwide [5,6,7]. Fungicide resistance in *B. cinerea* has unquestionably resulted in the decreased efficacy and even failure of the chemical control of gray mold. Therefore, it is necessary to develop new kinds of fungicides with high activity and new modes of action to solve the increasingly serious problem of resistance.

Among the plethora of various unique chemical structures, sulfonamides, the most common scaffolds in sulfur-containing molecules, have received considerable attention from all walks of life due to their eminent biological activities and serve an important function in the pharmaceutical industry [8,9]. In addition to medical applications, sulfonamides have also been registered as commercial pesticides (e.g., fungicides of flusulfamide, tolnifanide, cyazofamid, and amisulbrom, herbicides of pyroxsulam and penoxsulam, and the insecticide of fluorosulamid) and developed as pesticide candidates [10,11,12,13]. These results clearly demonstrate that developing sulfonamide pesticides with new structures and mechanisms is still highly desirable and promising. According to the pesticide registration information, only cyazofamid and amisulbrom have been registered and used in China [14]. Amisulbrom has been registered as a single fungicide. Cyazofamid has been widely used as a single fungicide or combined with other fungicides, including dimethomorph, fluopicolide, famoxadone, and pyraclostrobin [14].

**Figure 1:**
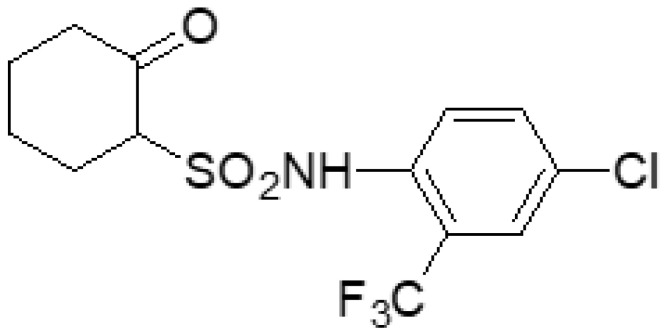
The chemical structure of **L13**.

During the screening of fungicide candidates, a series of sulfonamides with different scaffolds have been designed and synthesized with moderate to good fungicidal activities [15,16,17,18,19,20]. Among the sulfonamides developed by our group, *N*-(2-trifluoromethyl-4-chlorphenyl)-2-oxocyclohexyl sulfonamide (**L13**, generic name chesulfamide, codename CAUWL-2004-L-13, CAS No. 925234-67-9) (Figure 1) exhibited a broad spectrum of fungicidal activity and especially good fungicidal activity against *B. cinerea* with the EC_50_ of 2.12 μg/mL, comparable with the commercial fungicide procymidone (the EC_50_ value is 2.45 μg/mL) [18,21]. However, the mode of action of **L13** against *B. cinerea* is not clear, which deserves further investigation to be a commercial fungicide. Furthermore, the superior fungicidal activity against *B. cinerea* must be verified through pot culture and field efficacy trials.

In this work, the antifungal activity of **L13** against *B. cinerea* strains on tomatoes resistant to commercial fungicides has been studied. To elucidate the mode of action of **L13** against *B. cinerea*, the effects of **L13** on the ultrastructural of hyphae, electrolyte leakage, and respiration of mycelia suspension were explored. Furthermore, the evaluation of **L13** to control tomato gray mold (*B. cinerea*) through field efficacy trials and evaluation of the toxicology profile were reported. This research seeks to provide technical support and theoretical guidance for **L13** to become a real commercial fungicide.

## 2. Results and Discussion

### 2.1. Control Effect of L13 against B. cinerea Resistant to Commercial Fungicides

As mentioned above, with the frequent application of commercial fungicides, *B. cinerea* has developed resistance to many used fungicides under selective pressure. Thus, research and development of new fungicides with a new mode of action is highly desirable. In the present study, the control effect of **L13** against *B. cinerea* resistant to commercial fungicides of carbendazim, diethofencarb, and iprodione was investigated. Carbendazim is a widely-used broad-spectrum fungicide that inhibits mitotic microtubule formation and cell division [22], and the evolution of the resistance of *B. cinerea* to carbendazim in recent years has appeared [23]. Diethofencarb belongs to *N*-phenylcarbamates and can result in scattered nuclei and inhibited mitotic nuclear division [24], and has also developed resistance to *B. cinerea* in some regions [25]. Iprodione is an effective broad-spectrum, contact-type dicarboximide fungicide, commonly used to control early deciduous disease, inhibiting protein kinases and interfering with the intracellular signals of multiple cellular functions [26]. A study on the control effect of **L13** against *B. cinerea* is helpful to understand the mode of action and could provide a potential alternative to address the developed resistance of *B*. *cinerea* to these presently used commercial fungicides.

As shown in Table 1, the test fungicide, **L13,** gave good control against *B. cinerea* resistant to commercial fungicides of carbendazim, diethofencarb, and iprodione in the pot culture experiments. The control effect of **L13** against *B. cinerea* resistant to carbendazim reached 81.36% at a rate of 187.5 g. ai ha^−1^, and was better than carbendazim at a rate of 750 g. ai ha^−1^ (29.00%). The control effect of **L13** against *B. cinerea* resistant to diethofencarb reached 92.61% at a rate of 187.5 g. ai ha^−1^, and was better than diethofencarb at a rate of 267.9 g. ai ha^−1^ (76.36%). The control effect of **L13** against *B. cinerea* resistant to iprodione reached 78.27% at a rate of 187.5 g. ai ha^−1^, and was much better than iprodione at a rate of 375 g. ai ha^−1^ (2.36%). These results suggested that **L13** had a good control effect against *B. cinerea* and had no cross-resistance with the commercial fungicides carbendazim, diethofencarb, and iprodione.

### 2.2. The Mode of Action of L13 against B. cinerea

#### 2.2.1. Effect of L13 on Morphology and Ultrastructure Transformation of *B. cinerea*

**Figure 2:**
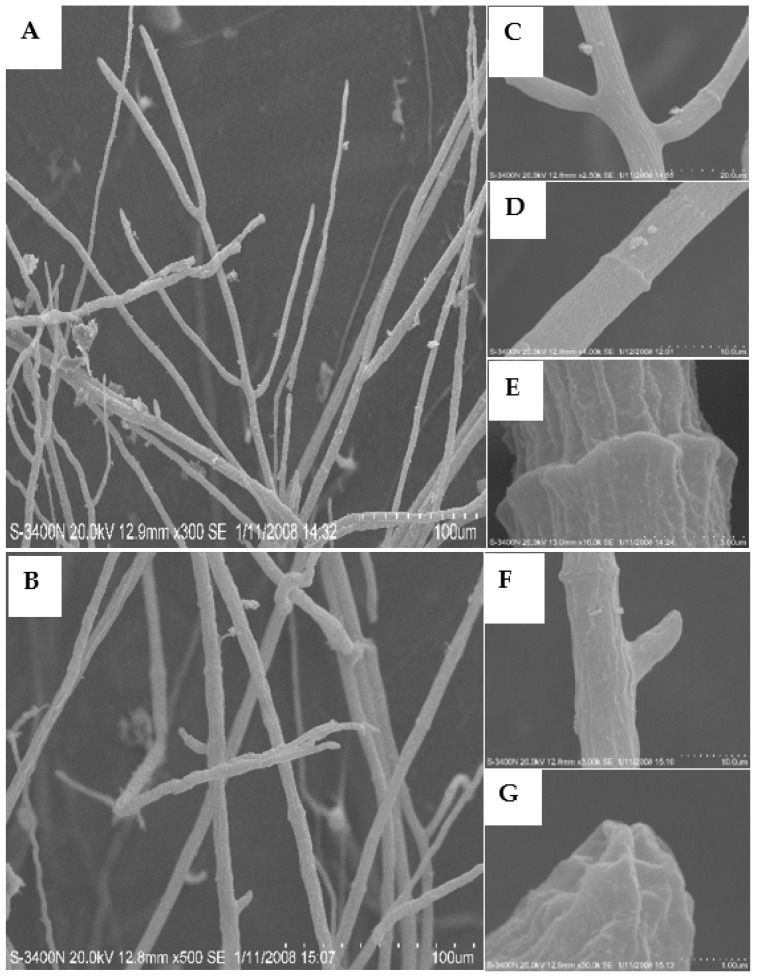
Scanning electron micrographs (SEM) of the hyphae from the colony of *B. cinerea* grown on PDA medium in the absence of **L13** (control). Scale bars: (**A**,**B**) 100.0 μm; (**C**) 20.0 μm; (**D**,**F**) 10.0 μm; (**E**) 3.0 μm; (**G**) 1.0 μm.

To investigate the mechanism by which **L13** affects the growth of *B. cinerea*, SEM and TEM were employed to examine the structural changes of mycelia after **L13** treatment. SEM images of the mycelia grown on the PDA without **L13** are shown in Figure 2. The mycelia were low density, fresh, and had a fine structure (Figure 2A). The interval between the two septa in hypha was relatively large (Figure 2B). The hyphae had a lot of branches that had a uniform distribution, and the surface of the hypha surface was evenly smooth (Figure 2C,D). The structure of the tip of the hypha and the septa were normal (Figure 2E–G). All of these images contain typical characteristics of *B. cinerea* [27].

**Figure 3:**
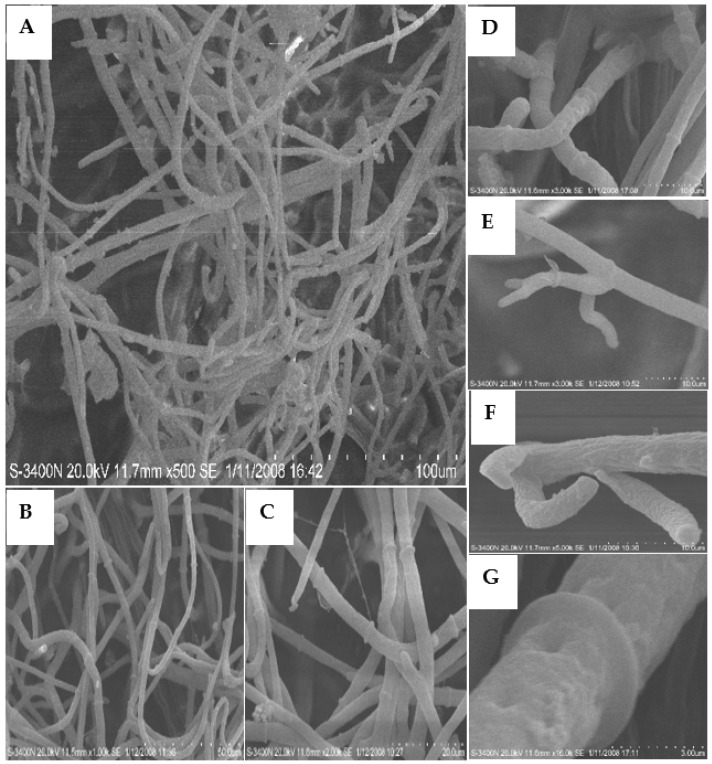
SEM images of the hyphae from the colony of *B. cinerea* grown on PDA medium with 50 μg/mL of **L13**. Scale bars: (**A**) 100.0 μm; (**B**) 50.0 μm; (**C**) 20.0 μm; (**D**–**F**) 10.0 μm; (**G**) 3.0 μm.

**Figure 4:**
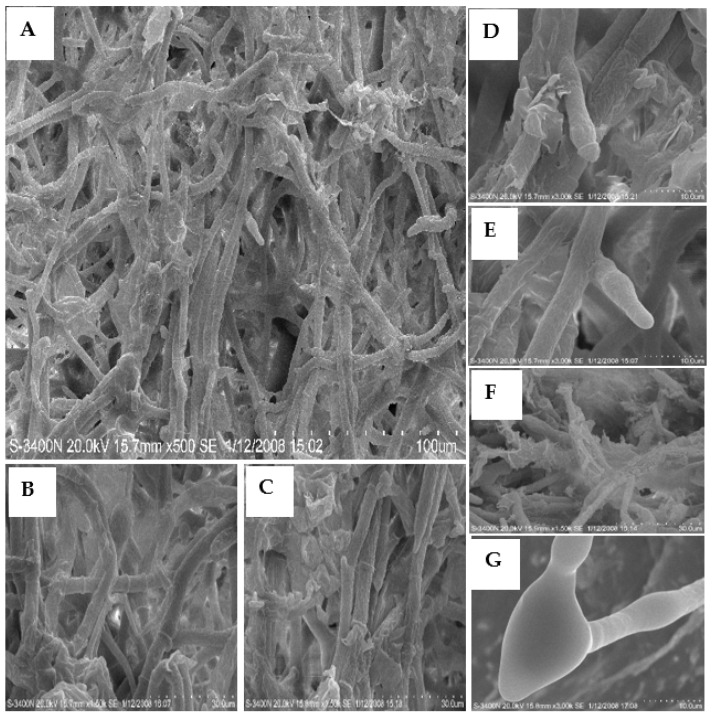
SEM images of the hyphae from the colony of *B. cinerea* grown on PDA medium with 100 μg/mL of **L13**. The mycelium swelled, parts of mycelia ruptured and formed abnormal configurations on the mycelium tip. Scale bars: (**A**) 100.0 μm; (**B**,**C**,**F**) 30.0 μm; (**D**,**E**,**G**) 10.0 μm.

SEM images of the mycelia grown on the PDA with 50 and 100 μg/mL of **L13** are shown in Figure 3 and Figure 4. After treatment with 50 μg/mL of **L13**, the growth of hyphae and its septa was similar to the blank controls (Figure 3A,B,F), but mycelial density increased significantly (Figure 3A,B). Meanwhile, the number of branches and septa of hyphae increased significantly (Figure 3A–C,E). The smoothy surface of the hypha surface was covered with many small shallow holes (Figure 3D,G). The structure of the tip of the hypha was deformed (Figure 3E,F). When treated with 100 μg/mL of **L13**, the morphological traits of mycelia showed a strong inhibitory effect (Figure 4). Mycelial density increased significantly, and some hyphae were ruptured (Figure 4A–C,F). The tendency to increase the number of branches and septa of hyphae was more obvious (Figure 4B,C). Moreover, there were irregular ramifications or abnormal configurations (contracted or swelling up) on the tip of the hypha (Figure 4D,E,G).

**Figure 5:**
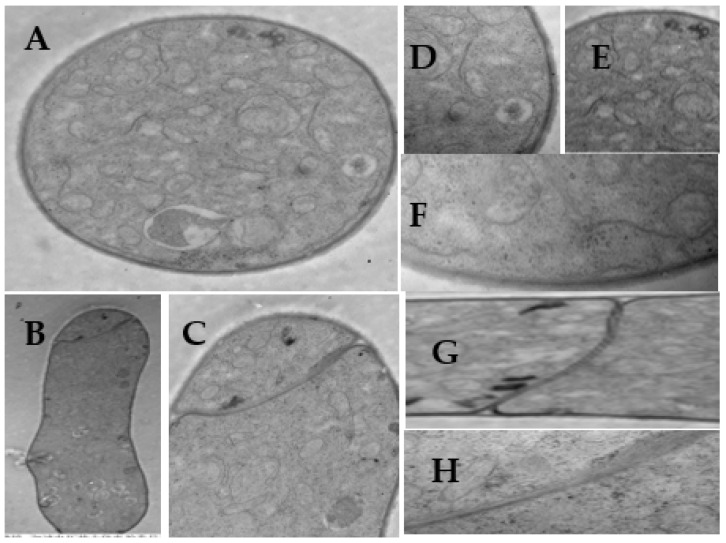
Transmission electron micrographs (TEM) of *B. cinerea* hyphae in the untreated control. Scale bars: (**A**,**B**,**G**,**H**) 1.0 μm; (**C**–**E**) 0.5 μm; (**F**) 0.1 μm.

**Figure 6:**
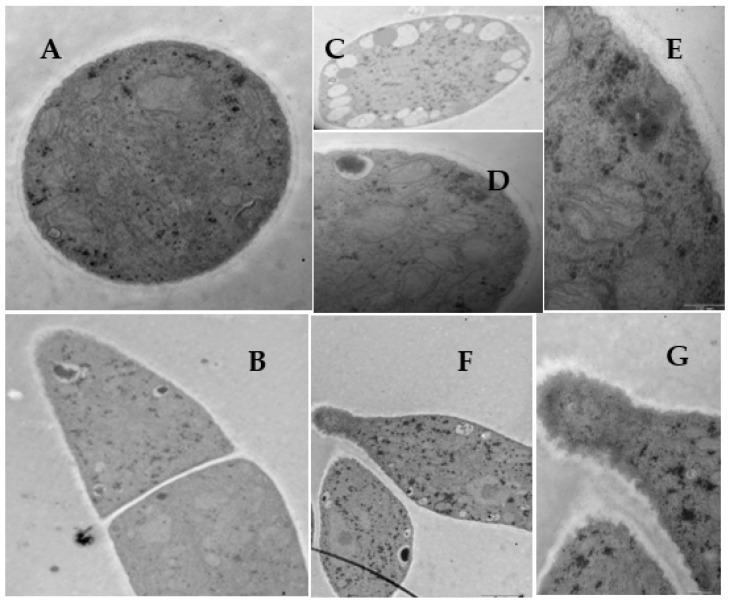
TEM images of *B. cinerea* hyphae with 50 μg/mL of **L13**. Scale bars: (**A**,**B**,**G**,**H**) 1.0 μm; (**C**–**E**) 0.5 μm; (**F**) 0.1 μm.

**Figure 7:**
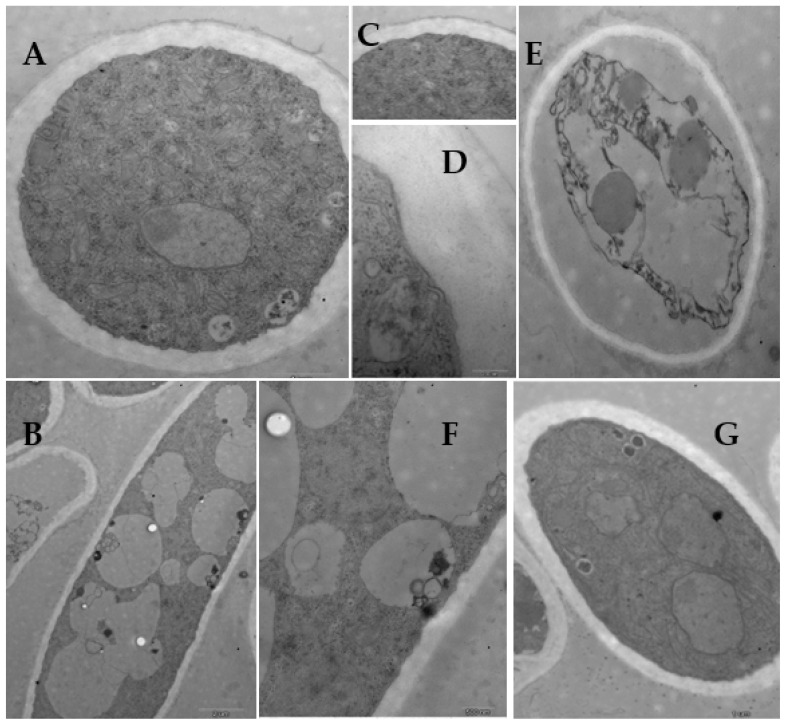
TEM images of *B. cinerea* hyphae with 100 μg/mL of **L13**. Scale bars: (**A**,**B**,**G**,**H**) 1.0 μm; (**C**–**E**) 0.5 μm; (**F**) 0.1 μm.

*B. cinerea* mycelial tips (5 mm) from the margins of the actively growing colony on the PDA medium were also examined by TEM (Figure 5, Figure 6 and Figure 7). The mycelia of *B. cinerea* grown in the absence of **L13** showed many of the cytological and ultrastructural features typical of vegetative hyphae of the genus [28] (Figure 5). The cell walls and septa of the hyphae were uniform. There were abundant organelles in the cytoplasm, such as the cell wall, vacuole, mitochondrial, endoplasmic reticulum, electron density, and lipid body.

In the presence of **L13** at 50 μg/mL, different ultrastructural modifications occurred in the hyphae (Figure 6). The amount of vacuole in the cell (Figure 6A,C,D) and electron density in the cytoplasm (Figure 6A,B,F) increased. The cell wall exhibited a decomposition trend (Figure 6E), and the tip of the hypha presented abnormal growth (Figure 6F,G). At a higher concentration (100 μg/mL), **L13** caused more conspicuous cytological changes (Figure 7). The striking characteristic was the cell wall decomposition (Figure 7A,C,D). The most extraordinary change in the intracellular is the organism disorder and the disappearance of most membrane-like structures, such as vacuole (Figure 7E,F).

SEM and TEM observations revealed that growth inhibition of *B. cinerea* as a response to **L13** was accompanied by obvious morphological and cytological changes, including excessive branching, irregular ramification or abnormal configuration (contracted or swelling up), and the decomposition of cell wall and vacuole. The cell wall of fungi is a sturdy structure providing physical protection and osmotic support, consisting of a complex of macromolecules with chitin, glucan, and mannose interconnected by covalent bonds. Hyphae growth, branching, cell fusion, and other morphogenetic events all depend on a balance between decomposition and extension of the hyphae wall and synthesis and the incorporation of new wall material [29,30]. In the present study, the hyphae walls of *B. cinerea* were decomposed, and excessive branching of the hyphae was observed, which is completely different from ergosterol biosynthesis inhibitor fungicides that inhibit the cell wall [30,31,32]. Another noteworthy alteration, however, was the disruption of the endomembrane system, especially vacuoles and secretory vesicles. The vacuole plays an important role in maintaining the fungal turgor pressure, and it is greatly important for mycelial growth. In this study, the vacuoles and vesicles related to cell wall synthesis [33] observed in control cells of *B. cinerea* were distorted and disrupted in **L13** treated cells. Hence, it may confirm that **L13** disrupts the endomembrane system indirectly by binding to the cell surface or directly from inside the cell.

#### 2.2.2. Effect of L13 on Mycelial Electrolyte Leakage of *B. cinerea*

**Figure 8:**
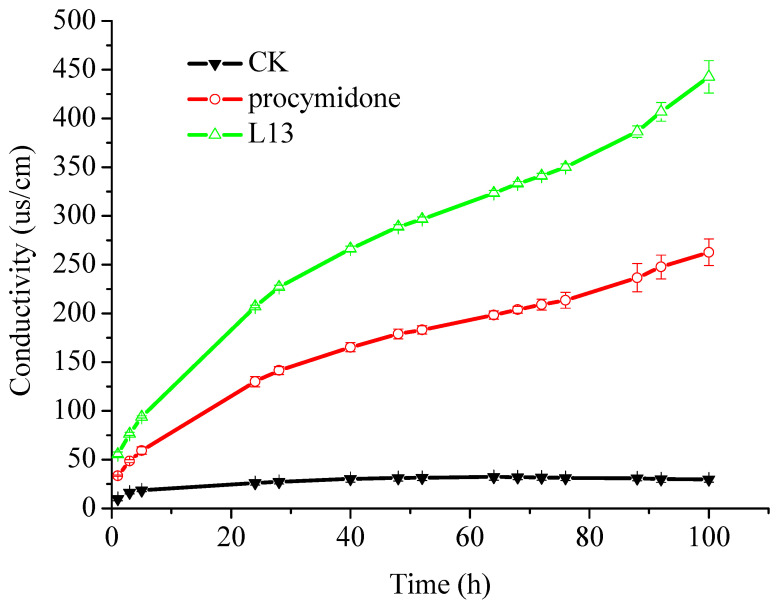
Electrolyte leakage from *B. cinerea* suspensions during different time exposure to different fungicides.

To examine the effect of **L13** on the cell membrane, the electrical conductivity of mycelia suspension was measured using commercial fungicides procymidone as a positive control (Figure 8). The conductivity of mycelia suspension treated with **L13** and procymidone greatly increased compared with the conductivity of the control mycelia at all treatment times. **L13** induced more significant electrolyte leakage from hyphae than procymidone. Thus, it was proposed that **L13** was related to the impairment of the cell membrane.

Electrolyte leakage is always used to indicate cell membrane permeability of hyphae exposed to various fungicides. The alteration of conductivity induced by **L13** was significantly greater than the alteration caused by procymidone which has been considered to cause some defects in the membrane but had no effect on the ion leakage or the water permeability [34]. The result indicated that **L13** caused certain damage to the mycelia cell membrane system, induced electrolyte leakage from the cell, and increased the conductivity of the solution. Thus, the decomposition of cell walls was most likely to be associated with biochemical changes in the plasmalemma induced by **L13**. We have reported that **L13** could reduce the content of the DNA and polysaccharide in the intact mycelia of *B. cinerea* [35], which provided a possible confirmation for the leakage of cellular content.

#### 2.2.3. Effect of L13 on Mycelial Respiration of *B. cinerea*

The effects of **L13** and famoxadone on the oxygen consumption of intact mycelia are shown in Table 2. **L13** almost did not affect the oxygen consumption of intact mycelia with 2.15% inhibition at 50 μg/mL and 17.44% inhibition at 100 μg/mL of **L13**, while famoxadone, a respiration inhibitor, exhibited a strong effect on the oxygen consumption of intact mycelia with 33.16% inhibition at 50 μg/mL and 60.42% inhibition at 100 μg/mL of famoxadone. These results proved that **L13** was not a respiration inhibitor and could not disturb the energy generation system of *B. cinerea.*

### 2.3. Control Effect of L13 against Tomato Gray Mold in Field Efficacy Trials

Gray mold caused by *B. cinerea* is a devastating disease on tomatoes, leading to severe economic losses in many countries worldwide [25]. The prevailing method to control gray mold is chemical fungicides applications [36]. Considering the commercial development of **L13**, field efficacy trials must be carried out under complex environmental scenarios, including the influences of sunlight, natural temperature and humidity, and environmental microorganisms. This is a process that must go through the commercialization of a pesticide. The good in vitro and pot test activity of **L13** against *B. cinerea* must be verified by field trials on tomatoes.

In two locations over 2 years, the control effect of **L13** against *B. cinerea* on tomatoes could reach 72–76% at a rate of 225 g ai ha^−1^ in Yuncheng city, which was better than the commercial fungicide iprodione 50% WP at the same dosage (Table 3). When the dosage of **L13** was 450 g ai ha^−1^, the field control effect could be up to 83%. In Gaocheng city, **L13** still demonstrated better control efficiency than iprodione 50% WP at the same dosage of 225 g ai ha^−1^, which was comparable to that in Yucheng city. The present field trials indicate that **L13** has great potential for commercial development.

### 2.4. Toxicological Test of L13

As shown in Table 4, the toxicological tests indicated that **L13** was a low toxicological compound [LD_50_ 1470 mg/kg·BW (male) and 2150 mg/kg·BW (female) for acute oral and LD_50_ > 2000 mg/kg·BW (male and female) for acute dermal] based on classification standard procedure of the People’s Republic of China. The teratogenesis, mutagenesis, and carcinogenesis tests were negative. Therefore, it can be concluded that **L13** is safe for rats and rabbits, which shows great potential for further commercial development.

## 3. Materials and Methods

### 3.1. Materials and Reagents

(*N*-(2-trifluoromethyl-4-chlorphenyl)-2-oxocyclohexyl sulfonamide) (**L13**) was prepared according to our previously reported method [18], and formulated as either a 50% (w/w) or 25% (w/w) WP (wettable powder) from **L13** (50% or 25%), naphthalenesulfonate (2%), lignosulfonate (5%), and white carbon black (43% or 68%), respectively. Carbendazim 50% WP was purchased from Jiangsu Xinyi Pesticide Factory (Xuzhou, China). Diethofencarb 50% WP was provided by the Institute of Vegetables and Flowers, Chinese Academy of Agricultural Sciences (Beijing, China). Iprodione 50% WP was obtained from Bayer Crop Science (China) Co., Ltd. (Hangzhou, China). Iprodione 25% SC was acquired from Jiangsu Kuaida Agrochemical Co., Ltd. (Nantong, China). All other chemicals were used as received without further purification.

The *B. cinerea* strains, which had resistance to carbendazim (HM-1), diethofencarb (HM-2), and iprodione (HM-3) for greenhouse pot studies, were provided by the Institute of Vegetables and Flowers, Chinese Academy of Agricultural Sciences (Beijing, China).

### 3.2. Greenhouse Test of L13 against B. cinerea on Tomato Resistant to Commercial Fungicides

The experiments were conducted in the greenhouse of the Institute of Vegetables and Flowers, Chinese Academy of Agricultural Sciences. The maximum and minimum temperatures of the greenhouse were 29 °C and 19 °C, respectively, and the average temperature was 24 °C. *B. cinerea* strains showing resistance to carbendazim, diethofencarb, and iprodione were cultivated on potato dextrose agar (PDA) plates for 4–5 d at room temperature. Tomato seeds (Ziyu) were sown in ceramic pots (300 mm in diameter) containing 0.6 kg of soil sterilized with formaldehyde (4–5 seeds for each pot). When plants were grown to the five-leaf stage, an aqueous solution of 25% **L13** WP (at dilution multiples of 1000 and 2000 times) was sprayed preventatively on the plant surface. Commercial fungicides of carbendazim 50% WP at dilution multiple of 500 times, diethofencarb 50% WP at dilution multiple of 1400 times, and iprodione 50% WP at dilution multiple of 1000 times were sprayed similarly on plant surface as positive controls, and equal amounts of water were sprayed as the blank control. The normalized water consumption per hectare was 750 L ha^−1^ for each treatment. After the application, the plants were inoculated with the *B. cinerea* strains. There were three replicates for each concentration. The severity of the disease was assessed according to a nine-grade classification at 7 d after inoculation [37], by which time the disease in the untreated control had fully developed.

### 3.3. Effect of L13 on Mycelial Ultrastructure of B. cinerea

*B. cinerea* mycelial tips (5 mm) from the margins of the actively growing colony on the PDA medium amended with 0, 50, and 100 μg/mL of **L13** were cut down after being cultured for 3 days. Then the mycelial tips were treated with 4% glutaraldehyde at 4 °C, then rinsed with 0.1 M phosphate buffer (pH 7.3) and fixed with 1% *w/v* osmium tetraoxide solution. After being rinsed with 0.1 M phosphate buffer three times, the mycelium block was dehydrated using a series of acetone solutions in the order of concentration: 30%, 50%, 70%, 80%, 90%, and anhydrous acetone. The processes of drying at a critical point, mounting, and gold spraying were completed at last and examined in a scanning electron microscope (S-3400N, Hitachi, Nissei Sanyo, Tokyo, apan) with an accelerating voltage of 18–20 kV.

The mycelial blocks were prepared according to the method given in Section 2.3. After dehydrating and embedding in Epon 112, thin sections were cut and double-stained with uranyl acetate and lead citrate. The grids were examined with a JEOL-1230 (JEOL, Tokyo, Japan) transmission electron microscope.

### 3.4. Effect of L13 on Mycelial Electrolyte Leakage of B. cinerea

Mycelial discs (5 mm in diameter) of *B. cinerea* grown on PDA plates were cut from the colony’s margins and placed in PDA liquid medium with 200 r/min shaking for 3 days. The mycelia in the culture medium were collected and washed three times by sterile distilled water and subsequently, filtered and weighed. A stock solution of **L13** and commercial fungicide procymidone as a positive control were diluted with sterile distilled water to 50 μg/mL, respectively. After adding 1.0 g of fresh mycelia into different solutions mentioned above, the conductivities of the solutions were measured using the DDS-11C model conductivity detector at 0, 1, 3, 5, 24, 28, 40, 48, 52, 64, 68, 72, 76, 88, 92, and 100 h after treatment. The experiment was repeated twice with three replicates per treatment.

### 3.5. Effect of L13 on Mycelial Respiration of B. cinerea

Mycelial plugs of *B. cinerea* from 3 days old colony margins were transferred to conical flasks containing 150 mL of PDA liquid medium for shake culture (25 °C, 150 r/min). After 5 days, mycelia were washed three times with 50 mM potassium phosphate buffer (pH 7.2) and resuspended in 0.1 M phosphate buffer (pH 7.2), containing 2% glucose to afford mycelial suspension at a concentration of 50 mg fresh weight of mycelia per milliliter. The mycelial suspension was treated with 50 and 100 μg/mL of **L13** and famoxadone, respectively. All measurements were carried out at 25 ± 1 °C. The ratio of the mycelial oxygen consumption was tested by oxygraphy (DW1, Hansatech Instruments Ltd., England), and the inhibition of respiration was calculated using the following formula:

I_R_ = (R_0_ − R_1_)/R_0_ × 100 (1)

I_R_: inhibition of respiration (%); R_0_, R_1_: the ratio of mycelial oxygen uptake pre and post addition of fungicides (nmol O_2_/min/mg mycelia).

### 3.6. Field Efficacy Trials of L13 against B. cinerea on Tomatoes

The field efficacy trials of **L13** against tomato gray mold were conducted in two locations of Yuncheng, Shanxi Province, and Gaocheng, Hebei Province. The experiments were conducted according to the National Agriculture Industry Standard of “Pesticide-guidelines for the field efficacy trials (I)-Fungicides against gray mold of vegetables” [38]. Tomato Jinpeng No. 1 was used as the experimental cultivar. There were six treatments in each trial, and the area of each plot was 5 × 3 m with four replications (24 plots).

When tomato plants were grown to the five-leaf stage, an aqueous solution of **L13** 50% WP at a rate of 450 and 225 g ai ha^−1^ was sprayed onto the plants using a hydraulic knapsack electric sprayer (CD-16B, Chaoda Instrument Co., Ltd., Taizhou, China) with a single cone nozzle operating at a typical working pressure 0.3 MPa. Iprodione 50% WP, or iprodione 25% SC at a rate of 225 and 562.5 g ai ha^−1^ were used as positive controls. Three applications were conducted at an interval of 7 d. The disease was assessed according to a nine-grade classification 7 d after the last application [37,38].

### 3.7. Toxicological Test of L13

The acute oral and dermal toxicity test on Wistar rats, eye and skin irritation test on rabbits, and Ames mutagenicity test were commissioned by the Institute for the Control of Chemical Toxicity in Tianjin. The bone marrow cell micronucleus assay and testis chromosomal aberration test of mice were commissioned by Tianjin Center for Disease Control and Prevention. All the toxicological tests were carried out according to the Toxicological Test Methods for Pesticide Registration (GB 15670-1995).

Based on the preliminary experiment, Horn’s method was used to determine the acute oral toxicity of **L13**. The dosage applied for rats was 464, 1000, 2150, and 4640 mg/kg·BW. For the acute dermal toxicity test, the dosage applied for rats was 2000 mg/kg·BW. For the eye and skin irritation test, rabbits with bodyweight from 2.5 to 3.0 kg were used, and 0.1 and 0.5 kg of **L13** was applied to the eyes and skin, respectively. For the Ames mutagenicity test, 0.05–1.0 mg per dish of **L13** was applied, and fenaminosulf (50 μg/dish), 2-aminofluorene (20 μg/dish), and 1,8-dihydroxyanthraquinone (50 μg/dish) were used as the positive controls. For the mouse bone marrow cell micronucleus assay, the dosages applied were 165, 82.5, 41.3, and 8.3 mg/kg·BW, and cyclophosphamide (60 mg/kg·BW) was used as a positive control. For the testis chromosomal aberration test of mice, the dosages applied were 206.2, 103.1, and 51.6 mg/kg·BW, and mitomycin C (2 mg/kg·BW) was used as a positive control.

### 3.8. Statistical Analysis

Statistical analysis was performed using the Origin software (OriginLab 2018, Northampton, MA, USA). One-way ANOVA was performed, followed by Duncan’s multiple range test. *p*-values < 0.05 were accepted as significant.

## 4. Conclusions

In this study, the antifungal activity of **L13** against *B. cinerea* resistant to commercial fungicides has been studied, and **L13** provided good control against *B. cinerea* resistant to commercial fungicides such as carbendazim, diethofencarb, and iprodione in the pot culture experiments. To elucidate the biochemical mode of action of **L13** against *B. cinerea*, the effects of **L13** on the ultrastructural of hyphae, electrolyte leakage, and respiration of mycelia suspension were explored. After treatment with **L13**, SEM observations showed that the mycelial density of *B. cinerea* was increased significantly, and some hyphae were ruptured. Moreover, there were irregular ramifications or abnormal configurations (contracted or swelling up) on the tip of the hypha. SEM observations demonstrated that at a higher concentration, **L13** caused more conspicuous cytological changes, especially concerning the decomposition of the cell wall, which may confirm that **L13** disrupted the endomembrane system indirectly by binding to the cell surface or directly from inside the cell. The alteration of conductivity induced by **L13** was greater than the alteration caused by procymidone as a positive control, implying that **L13** caused certain damage to the mycelia cell membrane system. Oxygen consumption of intact mycelia experiments proved that **L13** was not a respiration inhibitor. In two locations over 2 years, the field control effect of **L13** against tomato gray mold was better than the commercial fungicide iprodione at the same dosage. The toxicological tests indicated that **L13** is safe for rats and rabbits. The present studies preliminarily confirmed that **L13** has great potential for commercial development. Further research on the mode of action at the molecular level and field efficacy trials against more plant pathogens will be performed. As a common strategy to address the challenge of resistance, the sulfonamide **L13** can be combined with other fungicides with a different mode of action, such as dimethomorph, fluopicolide, famoxadone, and pyraclostrobin.

## Figures and Tables

**Table 1:** Control effect of **L13** against *B*. *cinerea* resistant to commercial fungicides.

Strains ^a^	Treatment	Dosage		Disease Area (mm)	Control Effect (%) ^c^
		Dilution Multiple ^b^	g.ai ha^−1^		
HM-1	**L13** 25%WP	1000	187.5	37.09	81.36 c
		2000	93.75	72.87	63.38 d
	Carbendazim 50% WP	500	750	141.29	29.00 e
	Diethofencarb 50% WP	1400	267.9	0.55	99.72 a
	Iprodione 50% WP	1000	375	15.88	92.02 b
	control	-	-	199.01	-
HM-2	**L13** 25%WP	1000	187.5	13.11	92.61 b
		2000	93.75	46.57	73.76 d
	Carbendazim 50% WP	500	750	1.79	98.99 a
	Diethofencarb 50% WP	1400	267.9	41.96	76.36 c
	Iprodione 50% WP	1000	375	2.16	98.78 a
	control	-	-	177.51	-
HM-3	**L13** 25%WP	1000	187.5	6.26	78.27 a
		2000	93.75	19.43	32.56 c
	Diethofencarb 50% WP	1400	267.9	11.64	59.60 b
	Iprodione 50% WP	1000	375	28.13	2.36 d
	control	-	-	28.81	-

^a^ HM-1 strains for *B*. *cinerea* resistant to carbendazim; HM-2 strains for *B*. *cinerea* resistant to diethofencarb; HM-3 strains for *B*. *cinerea* resistant to iprodione. ^b^ Dilution multiple means the volume of water (mL) used per unit mass (g) of a pesticide formulation. ^c^ Different letters (a, b, c, d) are significantly different according to Duncan’s multiple range test (*p* = 0.05).

**Table 2:** Respiratory inhibition of intact mycelia of *B. cinerea* by **L13**.

Inhibitors (μg/mL)	R_0_ (nmol O_2_/g/min)	R_1_ (nmol O_2_/g/min)	IR (%) *
**L13** (50)	173.51	169.93	2.15 d
**L13** (100)	173.51	143.48	17.44 c
Famoxadone (50)	173.51	115.99	33.16 b
Famoxadone (100)	173.51	68.79	60.42 a
CK	173.51	-	-

* Different letters (a, b, c, d) are significantly different according to Duncan’s multiple range test (*p* = 0.05).

**Table 3:** Control effect of **L13** against *B. cinerea* on tomatoes in field efficacy trials.

Treatment	Dosage		First Year		Second Year	
	Dilution Concentration	g.ai ha^−1^	Disease Index	Control Effect (%) *	Disease Index	Control Effect (%) *
Site: Yuncheng, Shanxi Province, China						
**L13** 50% WP	1000	450	2.87	82.85 a	2.78	83.32 a
**L13** 50% WP	2000	225	4.72	71.79 b	3.92	76.48 b
Iprodione 50% WP	2000	225	5.54	66.89 c	5.19	68.87 c
Untreated control	-	-	16.73	-	16.67	-
Site: Gaocheng, Hebei Province, China						
**L13** 50% WP	1000	450	2.40	71.66 ab	1.65	80.38 a
**L13** 50% WP	2000	225	2.83	66.59 b	1.71	79.67 a
Iprodione 25% SC	400	562.5	1.98	76.62 ab	2.15	71.22 a
Untreated control	-	-	8.47	-	8.41	-

* Different letters (a, b, c, d) are significantly different according to Duncan’s multiple range test (*p* = 0.05).

**Table 4:** Toxicological profile of **L13**.

Test Item	Value
Acute oral (rats)	LD_50_ 1470 mg/kg·BW (male); LD_50_ 2150 mg/kg·BW (female)
Acute dermal (rats)	LD_50_ > 2000 mg/kg·BW (male); LD_50_ > 2000 mg/kg·BW (female)
Eye irritation (rabbits)	No irritation
Skin irritation (rabbits)	No irritation
Teratogenesis	Negative
Mutagenesis	Negative
Carcinogenesis	Negative

## Data Availability

The data presented in this study are available in this article.
